# Medication Review with Follow-Up for End-Stage Renal Disease: Drug-Related Problems and Negative Outcomes Associated with Medication—A Systematic Review

**DOI:** 10.3390/jcm12155080

**Published:** 2023-08-02

**Authors:** Alfonso Pereira-Céspedes, Alberto Jiménez-Morales, Magdalena Palomares-Bayo, Fernando Martínez-Martínez, Miguel Ángel Calleja-Hernández

**Affiliations:** 1 Pharmaceutical Care Research Group, Pharmacy Faculty, University of Granada, 18071 Granada, Spain; alberto.jimenez.morales.sspa@juntadeandalucia.es (A.J.-M.); femartin@ugr.es (F.M.-M.); mangel.calleja.sspa@juntadeandalucia.es (M.Á.C.-H.); 2 Pharmacy Department, Hospital Universitario Virgen de las Nieves, 18014 Granada, Spain; 3 Centro Nacional de Información de Medicamentos, Instituto de Investigaciones Farmacéuticas, Pharmacy Faculty, University of Costa Rica, San José 11501-2060, Costa Rica; 4 Nephrology Department, Hospital Universitario Virgen de las Nieves, 18014 Granada, Spain; mapalomabayo@gmail.com; 5 Pharmacy Department, Hospital Universitario Virgen Macarena, 41009 Seville, Spain

**Keywords:** end-stage renal disease, medication review, drug-related problems, negative outcomes associated with medication

## Abstract

Background: This article reviews the available scientific literature on drug-related problems and negative outcomes associated with medications identified by medication review with follow-up for end-stage renal disease and discussed with the physicians. Methods: A systematic review was conducted of the scientific literature retrieved from the following databases: MEDLINE (via PubMed), Web of Science, SCOPUS, Cochrane Library: The Cochrane Central Register and Control Trials (CENTRAL) and Literatura Latinoamericana y del Caribe (LILACS), Medicina en Español (MEDES), and the SciELO bibliographic database (a collection of scientific journals). The following terms were used as descriptors and searched in free text: “end-stage renal disease”, “medication review”, “drug-related problems”, and “negative outcomes associated with medication”. The following limits were applied: “humans” and “adults (more than 18 years)”. Results: A total of 59 references were recovered and, after applying inclusion/exclusion criteria, 16 articles were selected. Of these selected articles, 15 provided information on drug-related problems and only 1 on negative outcomes associated with medications. Conclusions: It can be concluded that drug-related problems and negative outcomes associated with medications affect patients with end-stage renal disease, mainly those receiving renal replacement therapy. More evidence is needed, especially on negative outcomes associated with medication.

## 1. Introduction

Chronic kidney disease (CKD) has been recognized as a worldwide public health problem [1,2,3] that requires early detection and treatment to delay progression. When the disease progresses to a stage where kidney failure occurs, patients require renal replacement therapies, either by dialysis or by transplantation [1,2,4,5,6].

Regional differences in the prevalence of CKD have been documented around the world [7]. The globally estimated prevalence of CKD is 13.4% (11.7–15.1%), and the number of patients with end-stage renal disease (ESRD) needing renal replacement therapy is estimated to be between 4.902 and 7.083 million [1].

Chronic kidney disease is defined as abnormalities of the kidney structure or function present for more than 3 months with health implications. It has five stages according to the glomerular filtration rate (GFR). The end stage of chronic renal insufficiency is characterized by severe irreversible kidney damage (as measured by the level of proteinuria and the reduction of the GFR to less than 15 mL/min/1.73 m^2^). These patients generally require hemodialysis (HD), peritoneal dialysis (PD), or kidney transplantation (KT) [5].

Chronic kidney failure and ESRD are medically complex, require multiple medications to treat their various comorbidities [8], and generate high costs for the healthcare system. Patients with these conditions are at risk of drug-related problems [9,10,11,12,13,14,15,16,17,18,19,20,21,22,23,24,25,26,27,28,29,30] (DRPs) that may lead to increased morbidity, mortality, and costs [17].

A DRP is defined as an event or circumstance involving drug therapy that actually or potentially interferes with desired health outcomes [31,32].

A negative outcome associated with medication (NOM) is a result affecting the health of the patient that is or may be associated with the use of medications [32,33]. They can affect ESRD patients [8,34,35].

A medication review with follow-up (MRF) is a service in which the pharmacist evaluates the patient’s pharmacotherapy and intervenes in collaboration with the general practitioner and the patient themselves to ensure that the therapeutic goals are being achieved [36]. It is a structured evaluation of patient medications to optimize medication use and improve health outcomes, detect DRPs, and recommend interventions [37].

There are systematic reviews that focus on DRPs in stages 1–5 of CKD but do not focus on ESRD and MRF [2,10,38]. There is a lack of studies on NOMs in ESRD identified by MRF [29,35].

For all the reasons mentioned above, it seems essential to carry out a review of DRPs and NOMs identified by MRF in patients with ESRD.

Therefore, the objective of this article was to identify, evaluate, and summarize the findings of all relevant individual studies on DRPs and NOMs identified by MRF for patients with ESRD, making the available evidence more accessible to decision-makers.

## 2. Materials and Methods

### 2.1. Design and Procedure

A cross-sectional descriptive study and critical analysis of systematically retrieved works.

By reviewing the scientific literature, a systematic critical analysis of the relevant articles and gray literature was performed.

This systematic review was conducted based on “The PRISMA 2020 statement: an updated guideline for reporting systematic reviews” [39]. It is registered in the international prospective register of systematic reviews [PROSPERO: CRD42022324729] [40].

### 2.2. Source of Data Collection

All data were obtained by direct online consultation of the scientific literature in the following databases: MEDLINE (via PubMed), Web of Science, SCOPUS, Cochrane Library: The Cochrane Central Register and Control Trials (CENTRAL) and Literatura Latinoamericana y del Caribe (LILACS), Medicina en Español (MEDES), and the SciELO bibliographic database (a collection of scientific journals).

Journals, reference lists of the included studies, and previous scoping reviews related to DPRs and NOMs were searched to find additional studies. Other gray literature search engines, such as TESEO or DART for doctoral theses, were also used. The systematic review was supplemented by a comprehensive search of internet resources to identify gray literature on the subject, including websites specific to the research area, such as the Spanish Society of Nephrology, the Spanish Society of Hospital Pharmacy, the Spanish Society of Clinical and Community Pharmacy, and the Pharmaceutical Care Foundation (Spain).

### 2.3. Information Search

The thesaurus developed by the U.S. National Library of Medicine was referred to for the recovery of articles. No subject qualifiers (subheadings) were used nor were tag applications necessary.

The search strategy was planned around three domains:

- Population: adults with ESRD aged 18 years or older.
- Intervention: medication review.
- Outcome: DRPs and NOMs.

For this, the search syntax was generated using the Boolean intersection of three equations: (Equation (1)) and (Equation (2)) and (Equation (3)).

#### 2.3.1. Equation (1): “End-Stage Renal Disease”

“kidney failure, chronic”[MeSH Terms] OR “kidney failure chronic”[Title/Abstract] OR “ESRD”[Title/Abstract] OR “end stage renal failure”[Title/Abstract] OR “end stage renal disease”[Title/Abstract] OR “chronic kidney failure”[Title/Abstract] OR “end-stage kidney disease”[Title/Abstract]

#### 2.3.2. Equation (2): “Medication Review”

“medication review”[MeSH Terms] OR “medication review”[Title/Abstract] OR “medication reviews”[Title/Abstract] OR “review medication”[Title/Abstract] OR “reviews medication”[Title/Abstract]

#### 2.3.3. Equation (3): “Drug-Related Problems and Negative Outcomes Associated with Medication”

“drug related problems”[Title/Abstract] OR “drug therapy problems”[Title/Abstract] OR “medicines related problems”[Title/Abstract] OR “medication therapy problems”[Title/Abstract] OR OR (“negative outcomes associated with medication”[Title/Abstract] OR “medication-related”[All Fields] AND “negative outcomes”[Title/Abstract]) OR “outcomes associated with medication”[Title/Abstract]

The following filters (limits) were used: “Humans”, “Adults (more than 18 years)”. These filters were subsequently adapted for the databases mentioned above.

The final search equation was developed to be used in the MEDLINE database, via PubMed. Subsequently, this strategy was adapted to the characteristics of each of the other databases consulted and was completed by examining the bibliographic references of the selected articles to reduce the number of articles not recovered by the review.

The search was carried out from the first available database, according to the characteristics of each database, until 30 May 2023 (the time of the latest update).

Additionally, a search using a supplementary strategy was conducted to reduce the possibility of publication bias by searching the reference lists of relevant guidelines. Furthermore, experts in the domain were contacted by email to avoid missing relevant gray literature (materials and research produced by organizations outside the traditional commercial or academic publishing and distribution channels).

### 2.4. Study Selection

**Figure 1:**
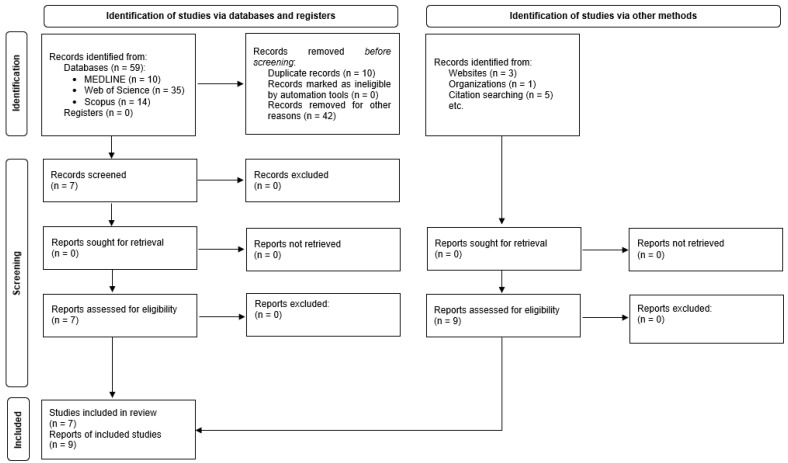
Results of search strategy and identification of publications included in the review.

The final selection of papers was made according to the following inclusion criteria: observational studies, original articles published in peer-reviewed journals, and pertinent works with available complete text, which had to be written in English, Portuguese, or Spanish (Figure 1).

The following were the exclusion criteria:

(1) Articles written in a language other than English, Portuguese, or Spanish.
(2) Articles without an abstract.
(3) Articles that do not mention any MRF in patients with ESRD.
(4) Articles that do not mention DRPs or NOMs in patients with ESRD.
(5) Articles mentioning patients under 18 years of age.
(6) Articles without a methods section, review articles, or case reports.

A selection of references was performed first on the basis of title and abstract and then after full-text review. Articles were selected based on the availability of the complete text. Any articles that did not meet the inclusion criteria were excluded.

### 2.5. Data Extraction

Two authors (A.P.C. and A.J.M.) assessed the suitability of the studies independently. For the selection process to be considered valid, it was established that the concordance between the two authors’ assessments (Kappa index) had to be higher than 0.60 (good or very good strength of concordance). Whenever this condition was met, any discrepancies were resolved by consulting the third author (M.A.C.H.), and subsequently by consensus among all the authors.

Double-entry tables were used to check the extracted data; this made it possible to detect errors and correct them by re-consulting the original documents. 

### 2.6. Study Variables

Articles were collected according to study variables to systematize and facilitate comprehension of the results. The following data were considered:

- Author, year, and country: first author of the article selected, year of publication of the article, and location where the study took place.
- Study design and duration: procedures, methods, and techniques through which the article was accepted for review. Duration of the study.
- Population studied: adults with ESRD (age, ethnicity, sex).
- Study aim: objective or aim of the study.
- DRPs: Total, type, and frequency of DRPs.
- NOMs: Total, type, and frequency of NOMs.
- Pharmacist interventions: Total and relevant findings of pharmacist interventions related to DRPs and NOMs.
- Types of medication most commonly associated with DRPs/NOMs.

### 2.7. Methodological Quality Assessment

The quality of the selected articles was assessed using the STROBE (Strengthening the Reporting of Observational Studies in Epidemiology) 45 checklist as support [41]. This contains 22 essential items that should be included in the reporting of observational studies. 

A score of 1 or 0 was recorded for each item according to whether or not the article met that criterion. In the event that evaluation of a particular item was not necessary, no score was assigned for that item, and it was recorded as not applicable (NA). When an item was composed of several points, these were evaluated independently, giving each the same weight, and the final result for that item was the average of these separate scores, so that in no case could the score exceed 1 point per item.

## 3. Results

With the search criteria described, 59 references were retrieved: 35 from the Web of Science (59.3%), 14 from Scopus (23.7%), and 10 from MEDLINE (19.9%). No references were retrieved from the Cochrane Library Literatura Latinoamericana y del Caribe (LILACS), Medicina en Español (MEDES), or the SciELO bibliographic database (collection of scientific journals).

Nine additional studies were identified from other sources (manual search and contribution by experts) [29,34,42,43,44,45,46,47,48]. 

After the elimination of duplicates, application of the inclusion and exclusion criteria, consultation of the bibliographies of the selected articles, and consultation with experts (Figure 1), 16 documents with full text available were selected and retrieved [20,29,34,42,43,44,45,46,47,48,49,50,51,52,53,54]. One of them was a doctoral thesis [34] and was related to another selected study [29]. The characteristics of the studies are summarized in Table 1.

The concordance between the evaluators on the relevance of the articles was 100%.

### 3.1. Study Characteristics and Quality

The 16 studies identified were carried out in the U.S.A. (n = 6), Spain (n = 2), Singapore (n = 2), Indonesia (n = 2), and Saudi Arabia, Taiwan, New Zealand, and India, with 1 study each. Most of the articles reviewed were written in English, except two that were written in Spanish [29,34]. 

Of the papers reviewed, 12 were observational studies [42,43,44,45,46,47,48,49,51,52,53,54] (10 cross-sectional and 2 cohort [45,53]). In addition, two were clinical trials [20,50] (one of which was controlled randomly selected [20]) and two had a quasi-experimental design [29,34]. The follow-up time ranged from 1 to 18 months. 

The studies were carried out in various health care settings: hospitals [29,34,42,44,45,52], HD units [20,46,47,48,49,51,53,54] and KT units [43,50].

Articles included patients receiving kidney replacement therapy: from available data, 841 patients were treated with HD [20,29,34,42,44,45,46,47,48,49,51,52,53,54], 253 with KT [29,34,43,50] and 6 with PD [42].

The patients included in the studies were aged 49 to 66 years on average; three studies did not mention the age of the patients [43,44,46].

There were 12 studies that included both male and female patients; 2 did not mention the patients’ sex [43,46].

The studies included patients of a range of ethnicities, at various frequencies. However, 10 of the studies reviewed did not mention ethnicity [29,34,43,44,45,46,47,48,49,50].

### 3.2. Medication Review with Follow-Up Method

With regard to the MRF method used, 14 studies followed a specific clinical methodology for MRF and two studies [29,34] followed the Dáder Method developed by the Pharmaceutical Care Research Group at the University of Granada [32].

### 3.3. Drug-Related Problems

From the data available, a total of 10250 DRPs were identified and classified in 15 of the studies [20,29,42,43,44,45,46,47,48,49,50,51,52,53,54]. The DRPs were of the following kinds: wrongly administered drug [29,52], inappropriate dose (underdose or overdose), dosage schedule, and/or duration [20,42,43,44,45,46,47,48,50,51,52,53], duplication [29,42,47,52], non-adherence [29,51,52,54], drug interactions [29,42,44,47,49,52,54], ADRs [20,29,42,43,44,45,48,50,52,53,54], health problem not adequately treated [20,43,44,47,50,51,52,54], and others [42,43,44,45,46,47,48,50,52,54]. 

With regard to the DRP classification system, seven studies [20,42,43,47,50,51,54] used Strand et al. [55], one study [53] used Medi-Span (Wolters Kluwer), one study [29] used the Granada Third Consensus [32], and two studies [45,48] used the Pharmaceutical Care Network Europe classification system [31]. However, four studies did not mention the DRP classification system used [44,46,49,52].

Four studies [29,34,43,50] mentioned DRPs most commonly involved in KT (wrongly administered drug [29], inappropriate dose (underdose or overdose), dosage schedule and/or duration [43,50], non-adherence [29], drug interactions [29], ADRs [29,43,50], health problems not adequately treated [43,50] and others [50].

Ten studies [20,29,42,43,47,48,50,51,53,54] consistently identified the medication classes most commonly associated with DRPs to be cardiovascular medications, immunosuppressants, and medications for anemia and mineral bone disorders. Moreover, in the specific population of KT patients, immunosuppressants emerged as notably frequent contributors to DRPs, as reported by study [32].

Two of the papers reviewed [48,51] mentioned factors for multiple DRPs: ethnicity [51], length of time on dialysis [51], age [51], comorbidities [48], and number of medications prescribed [48].

### 3.4. Negative Outcomes Associated with Medication

Only one study related to cinacalcet (mineral bone disease medication) identified 9 NOMs [34,35] in 34 ESRD patients, according to the Granada Third Consensus [32], as follows: the patient suffers a health problem associated with a non-quantitative safety problem of the medication (n = 5), the patient suffers a health problem associated with quantitative ineffectiveness of the medication (n = 2), the patient suffers a health problem associated with a quantitative safety problem of the medication (n = 1), and the patient suffers a health problem as a consequence of not receiving the medicine that they need (n = 1) or an untreated health problem.

The same study showed that one KT patient (n = 1) suffers a health problem associated with quantitative safety problem of the medication.

### 3.5. Pharmacist’s Interventions

Eleven studies [29,34,43,45,46,47,48,49,50,52,54] mentioned pharmacist interventions related to resolved DRPs or NOMs highly accepted by the patient or the physician. From the available data, a total of 3153 interventions were made.

### 3.6. Quality

The scores achieved in the quality assessment of the selected articles using the STROBE questionnaire ranged between 11 and 22 (Table 2).

Five of the journals involved, in which eight of the articles reviewed were published [20,42,43,46,49,50,53,54], are listed in the Journal Citation Report (JCR). Three, containing three of the articles reviewed [29,45,47], are listed in the Emerging Source Citation Index (ESCI) of the JCR. However, four journals containing four of the articles reviewed [44,48,51,52] are not listed in the JCR or the ESCI. One of the studies is a doctoral thesis [34].

## 4. Discussion

This systematic review provides relevant findings on DRPs and NOMs in ESRD. From the information analyzed, it can be seen that DRPs are a real health issue affecting people with CKD in its different stages, especially those undergoing renal replacement therapy, as their polymedication and multi-pathology characteristics [2,34,38] increase as CKD evolves, leading to NOMs (manifest or potential).

Although NOMs are important in clinical practice, few studies have been published [34], opening up a field of research in this area that can support clinical practice, including patient-centered models.

The main DRPs identified in ESRD (HD, PD or KT) were wrongly administered drugs [29,52], inappropriate dose (underdose or overdose), dosage schedule, and/or duration [20,42,43,44,45,46,47,48,50,51,52,53], duplication [29,42,47,52], nonadherence [29,51,52,54], drug-drug interactions [29,42,44,47,49,52,54], probably due to ADRs [20,29,42,43,44,45,48,50,52,53,54], health problems insufficiently treated [20,43,44,47,50,51,52,54], and others [42,43,44,45,46,47,48,50,52,54].

In terms of NOMs in ESRD [34,35], the following stand out: untreated health problems, quantitative and non-quantitative ineffectiveness, and quantitative and non-quantitative safety.

In the context of DRPs most commonly associated with KT, the identified issues encompass the administration of the wrong medication [29], inappropriate dosing (underdose or overdose), deviations in dosage schedule and/or duration [43,50], patient non-adherence to prescribed regimens [29], drug interactions [29], ADRs [29,43,50], inadequate treatment of health problems [43,50], and other contributing factors [50].

Conversely, the NOMs observed in KT primarily revolved around non-quantitative safety [29,34].

The recognition of these common DRPs and NOMs in the context of HD, PD, or KT is crucial for healthcare practitioners and policymakers alike. These findings serve as a valuable foundation for implementing targeted interventions, including pharmacist-led interventions and MRF to enhance medication safety and optimize patient care outcomes in ESRD.

Through a comprehensive understanding of the prevalent DRPs and NOMs, healthcare professionals can tailor their approaches to minimize potential risks and maximize the benefits of medication therapies in the complex setting of ESRD. Continuous efforts to address these challenges can lead to improved patient experiences and overall treatment success in this population.

The findings from several studies [20,29,42,43,47,48,50,51,53,54] consistently indicate that immunosuppressants, medications for mineral bone disorders, and antianemic preparations are among the most frequently associated with DRPs. Moreover, these same classes of medications, namely immunosuppressants and medications for mineral bone disorders, emerge as the most common medications associated with NOMs [29,34].

The results supporting the link between these specific medication classes and DRPs and NOMs highlight their critical role in MRF for patients undergoing immunosuppression and mineral bone disorders. These findings underscore the importance of MRF, and pharmacist-led interventions to mitigate potential adverse effects and optimize patient outcomes in clinical settings.

This review has certain possible limitations. The design of the cross-sectional and cohort studies reviewed [45,53] provides an evidence level and a recommendation grade of IIb and III, according to the U.S. Agency for Health Research and Quality. However, the topic of study must be considered as DRPs and NOMs, and consequently, it must be assumed that it is probably not possible to aspire to a high design and recommendation grade [8,38].

Although systematic reviews must be based on observational studies and designs that guarantee stronger scientific evidence, in this analysis all the studies that focused on the subject topic were included.

The real limitations of this review are those of each study: for example, the number of medications per patient, study duration, and acceptance rate for pharmacist interventions in Alshamrani et al. [47], and the small sample size and lack of control group in the case of Mirkov [51]. In addition, the retrospective analysis of observational data and descriptive statistics by Daifi et al. [54] potentially involves residual confounding bias, and Chen’s study [52] also has several limitations (a single-site study with small sample size).

The methodological quality of the studies available in this systematic review and the heterogeneity of the studies evaluated, limit the possibility of meta-analysis.

In this systematic review, one of the limitations lies in the decision to include only studies conducted in English and Spanish, thereby excluding research published in other languages. This limitation stems from practical considerations and resource constraints during the literature search and review process.

By restricting the review to English and Spanish studies, there is a potential for language bias, as relevant studies conducted in other languages might have been overlooked. Consequently, this may limit the comprehensiveness and generalizability of the findings. Studies in different languages could provide valuable insights and diverse perspectives on the topic under investigation.

Moreover, language restriction may lead to the exclusion of certain regions or countries where research in ESRD and DRPs could be prevalent. This may inadvertently affect the representativeness of the study and the applicability of the findings to a broader international or multicultural context.

Furthermore, the decision to limit the language of included studies could affect the identification of rare or niche findings that might be present in research published in less common languages. These unique findings could potentially contribute to a more nuanced understanding of DRPs and NOMs in ESRD patients.

In future iterations of similar systematic reviews, it would be beneficial to consider overcoming this limitation by incorporating the resources and expertise necessary to search and include studies in a broader range of languages. Additionally, collaborating with experts who are proficient in various languages may facilitate the identification and inclusion of relevant non-English and non-Spanish studies, thereby enhancing the overall rigor and comprehensiveness of the review.

In addition to providing an opportunity to describe the NOMs and DRPs most frequently found in this population, this systematic review may raise awareness among healthcare professionals to help them identify such problems in clinical practice. Negative outcomes associated with medication, as well as their causes or DRPs, can be detected and resolved through physician-pharmacist collaboration, with the aid of MRF.

In addition, this review encourages further study of the clinical, human, and economic impact of NOMs on ESRD in clinical practice, the collaboration of nephrologists and pharmacists, and the methodological quality of research.

## 5. Conclusions

In conclusion, our systematic review reveals that DRPs are prevalent in approximately 9.32% of ESRD patients, while the NOMs impact is approximately 26.47% of this patient population, particularly those undergoing renal replacement therapy. Among the medication classes implicated in these issues, cardiovascular medications, immunosuppressants, and medications for anemia and mineral bone disease were found to be the most commonly involved.

While our findings expose the significance of DRPs and NOMs in ESRD patients, it is crucial to acknowledge that further research is required to enhance our understanding of NOMs, particularly in this context. Additional evidence would greatly contribute to developing more targeted interventions and strategies to mitigate the negative outcomes of medications in ESRD patients effectively.

In light of the limitations and complexities identified in our study, we believe that ongoing efforts to investigate and address DRPs and NOMs are warranted, as they hold substantial implications for optimizing medication review and patient outcomes in this population.

## Figures and Tables

**Table 1:** Characteristics of the studies included in the systematic review.

			Participants, Demographics					DRPs		NOMs		Pharmacist Interventions		
First Author, Year, Country, Ref. No.	Study Design (Duration)	Study Setting	N (at Baseline)	Sex n (%)	Age Mean (SD)	Ethnicity (%)	Aim	Total (n)	Type and Frequency (%)	Total (n)	Type and Frequency. (%)	Total (n)	Relevant Findings	Types of Medication Most Commonly Associated with DRPs/NOMs
Grabe 1997 U.S.A. [49]	Observational retrospective (1 month)	Outpatient HD unit	45 HD	24 (53.3) male, 21 (46.7) female.	52 (16)	Not reported	To identify DRPs in hemodialysis outpatients by performing medication reviews, make appropriate recommendations, determine the significance of any interventions, and estimate outcome in terms of any changes in number of medications/patient or doses/day.	126	Drug interactions (27.5).	Not reported	Not reported	102	Most of the interventions were significant and possibly led to better therapeutic outcomes.	Not reported
Possidente 1999 U.S.A. [42]	Observational prospective (1 month)	University teaching hospital	37 (31 HD, 6 PD)	19 (51.4) male, 18 (48.7) female	65.9 (12.7)	Caucasian (97.3), Hispanic (2.7)	To evaluate the continuity of drug therapy and identify and resolve DRPs during the complete hospitalization process in patients receiving long-term dialysis.	161	Failure to receive a prescribed drug (41.0), problems related to drug dosage, either overdosage or underdosage (25.5), drug interactions (1.9), therapeutic duplication (2.5), and ADRs (1.2)	Not reported	Not reported	Not reported	Physicians agreed with 96% of the pharmacist recommendations, indicating strong support for pharmacist assistance in monitoring drug therapy.	Medications for mineral bone disorder, antianemic preparations, and anti-infectives
Chisholm 2000 U.S.A. [43]	Observational prospective (18 months)	Ambulatory care RT	201 KT	Not reported	Not reported	Not reported	To document the number and types of recommendations made by a pharmacist to the multidisciplinary renal transplant team, to determine the rate of acceptance of the recommendations, and to determine the potential impact of the recommendations on patient care.	811	Untreated indication (28.4), overdose (26.6), subtherapeutic dosage (18.1), medication use without an indication (10.1), ADRs (7.6), improper medication selection (7.0), failure to receive medication (2.1).	Not reported	Not reported	844	96% (n = 811) were accepted. Nearly all (99%) of the accepted recommendations were judged to have a significant, very significant, or extremely significant potential impact on patient care	Immunosuppressants and cardiovascular medications
Manley 2003 U.S.A. [20]	Clinical trial randomly selected controlled (10 months)	Non-profit outpatient dialysis unit	133 HD (66 pharmaceutical care group; 79 usual care group).	74 (55.6) male, 59 (44.4) female	62.8 (15.0)	Black (78.2), Caucasian (17.3), other (4.5)	To determine the rate, number, type, severity, and appearance of DRPs, as identified through pharmaceutical care activities, in patients with ambulatory HD.	354	Medication dosing problems (33.5), ADRs (20.7), and an indication that was not currently being treated (13.5)	Not reported	Not reported	Not reported	Not reported	Cardiovascular medications (29.7%), endocrine medications (15.5%), and specific medications (medications for mineral bone disorder, antianemic preparations) (15%)
Chua 2003 Singapore [46]	Observational prospective (3 months)	HD center	31 HD	Not reported	Not reported	Not reported	To identify DRPs in HD patients, intervene, and resolve them.	83	Drug underdose (35)	Not reported	Not reported	73	62% of the accepted recommendations were classified as significant	Not reported
Wang 2008 Taiwan [50]	Clinical trial enrolled subjects uncontrolled (15 months)	RT clinics	37 KT	20 (54.1) female, 17 (45.9) male	Not reported Age range 22-60 years	Not reported	To investigate the effects on treatment results of clinical pharmacists joining RT clinics to provide pharmaceutical care.	55	Medication selection (84.5), improper laboratory data (12.7), dosage adjustment (14.5), ADRs (10.9), untreated indications and medication use without an Indication (9.1), failure to receive medication (5.5), other (3.6)	Not reported	Not reported	55	81.8% were classified as clinically significant. The mean acceptance rate of physicians for the types of recommendation was 96.0%	Cardiovascular medications (32.6%), immunosuppressants (23.9%), and antimetabolites (26.1%)
Mirkov 2009 New Zealand [51]	Observational prospective (7 months)	HD units	64 HD	39 (60.9) female, 25 (39.1) male	65 (Not reported) Age range 24–82 years	Pacific People (46.9), New Zealand Maori (25), European (20.3), Other (7.8)	To implement the pharmacist medication review clinic and establish a sustainable clinical pharmacy service.	278	Non-adherence to medication regimen (33.0), medication requiring dose decrease (9.3), indication requiring new medication (8.6).	Not reported	Not reported	493	Not reported	Not reported
Chemello 2012 Spain [29,34]	Quasi-experimental pre-post-intervention study (12 months)	Hospital	34 (19 HD, 15 KT)	17 (50.0) female, 17 (50.0) male	51.5 (12.4)	Not reported	To assess the effect of pharmaceutical intervention on the identification of DRPs, improve desired clinical outcomes, and evaluate the effectiveness of cinacalcet in achieving clinical outcomes recommended by the Kidney Disease Outcomes Quality Initiative (KDOQI) Clinical Guidelines.	29	Non adherence (51.7), ADRs (13.8), Drug interaction (3.5), therapeutic duplication (3.5), wrong dosage administered (6.9)	9	Untreated health problem (11.1), quantitative ineffectiveness (22.2), non-quantitative safety problem (55.6), quantitative safety problem. (11.1)	34	After the intervention, 9 drug-related problems remained, which means that 68.9% of them were resolved (*p* < 0.001), reaching an adherence of 80%. Parathyroid hormone, calcium and calcium-phosphorus product serum levels decreased significantly after 3 months of treatment (*p* < 0.001, <0.001 and 0.045, respectively), achieving the KDOQI Clinical Guideline recommendations.	Cinecalcet
Chen 2013 Singapore [52]	Observational prospective (5 months)	General hospital	30 HD	15 (50.0) female, 15 (50.0) male	62.3 (10.0)	Chinese (73.3)	To evaluate the prevalence of DRPs identified and the types of interventions made by pharmacists.	94	Drugs with no indication (2,1), therapeutic duplication (8.5), untreated indication (14.9), improper selection of drugs (5.3), overdose of drugs (9.6), underdose of drugs (1.1), drug-drug interactions (1.1), drug-food interactions (1.1), non-adherence (41.5), ADRs (11.7), administration issues (3.2).	Not reported	Not reported	54	Almost half involved suggestions to modify dosing regimens (51.9%), followed by suggestions to add new drugs (16.7%) and to increase the doses, discontinue drugs (13.0%). Total DRPs solved (%): 63 (67.0%)	Not reported
George 2017 India [44]	Observational prospective (6 months)	Hospital	79 HD	56 (70.9) male, 23 (29.1) female	Not reported	Not reported	To determine DRPs in HD patients.	301	Drug interactions (86.4), ADRs (5.0), indication without drug therapy (4.0), improper drug selection (1.3), overdose (3.0), failure to receive drug (0.3).	Not reported	Not reported	Not reported	Not reported	Not reported
Lumbantobing 2017 Indonesia [48]	Observational prospective (5 months)	HD unit	86 HD	45 (52.3) male, 41 (47.6) female	Not reported Age range 41-60 years	Not reported	To identify DRPs and assess the effect of pharmacist intervention on the number and types of DRPs in HD outpatients	337	Failed therapy (18.7), suboptimal therapeutic effects (52.2), indications of non-administration of drugs (2.4), non-allergic adverse drug effects (26.7).	Not reported	Not reported	277	Not reported	Calcium carbonate, ferrous sulfate, erythropoiesis-stimulating agents, and omeprazole
Alshamrani 2018 Saudi Arabia [47]	Observational prospective cross-sectional (2 months)	Outpatient HD unit	83 HD	42 (51) males, 41 (49.4) female	63 (not reported)	Not reported	To determine the prevalence of polypharmacy and DRPs in HD patients	280	Medication use without indication (36.0), subtherapeutic dosing (23.0), overdosing (15.0), deprescribing of medication (41.0), medication use without indication (89.0), duplicate therapy (11.0), drug interaction (n = 184)	Not reported	Not reported	280	Not reported	Medications for gastrointestinal or acid-related disorders, cardiovascular medications, and antidepressants
Manley 2020 U.S.A. [53]	Retrospective cohort study (12 months)	Dialysis clinics	726 ESRD HD (89%)	334 (46) female, 392 (54) male	64 (15)	White (46), black (43), other (4), unknown (8)	Not reported	5466 potential	Medication dosing issues (31.0), comprising “dose too high” (22.0) and “dose too low” (9.0), actual or potential ADRs (29.0), unnecessary drug therapy (17.0).	Not reported	Not reported	Not reported	Not reported	Cardiovascular medications, medications for gastrointestinal or acid-related disorders, analgesics and endocrine and metabolic medications.
Daifi 2021 U.S.A [54]	Retrospective (14 months)	HD facilities	157 HD	81 (52) female, 76 (48) male	63.0 (not reported) Age range: 26–92 years	African American (79), White (4), Hispanic (6), other (11)	To evaluate the impact of a clinical pharmacist in an HD facility by assessing the efficacy of medication reconciliation in HD patients and evaluating the potential impact on the health system through estimated cost avoidance.	1407	Non-adherence (31.0), ADRs (2.6), dose too high (4.6), dose too low (13.1), needs additional drug therapy (21.5), unnecessary drug therapy (8.8), wrong dose (4.5), additional/ other DRP (0.6), drug-drug interaction (1.1), cost, accessibility, refills (11.9).	Not reported	Not reported	964	Not reported	Antihypertensives, vitamin D analogues and calcimimetics.
Peri 2022 Indonesia [45]	Analytical cohort study (6 months)	Hospital	83 HD	54 (65) male, 29 (35) female	48.91 (not reported) Age range 11–61 years	Not reported	To analyze the impacts of pharmacy interventions on DRPs, blood pressure, and quality of life in HD	470	Sub-optimal drug effects (50.85), untreated symptoms (22.1), no drug effect (8.9), ADRs (17.7)	Not reported	Not reported	470	Not reported	Not reported

RT: replacement therapy, HD: hemodialysis, PD: peritoneal dialysis, KT: kidney transplantation, DRP: drug-related problem, NOM: negative outcome associated with medication, ADR: adverse drug reaction, n: number.

**Table 2:** Quality of the methodology of the studies according to the 22-point STROBE guide assessment.

	Questionnaire Elements																							
First Author, Year, Country, Ref. No.	1	2	3	4	5	6	7	8	9	10	11	12	13	14	15	16	17	18	19	20	21	22	Total	%
Grabe 1997 U.S.A. [49]	1	1	1	1	1	1	1	1	1	1	1	1	1	1	1	1	1	1	1	1	1	0	21	95.45
Possidente 1999 U.S.A. [42]	1	1	1	1	1	1	1	1	1	1	1	1	1	1	1	1	1	1	0	1	0	0	19	83.36
Chisholm 2000 U.S.A. [43]	1	1	1	1	1	1	1	1	0	1	1	0	1	0	1	1	1	1	0	1	0	0	16	72.73
Manley 2003 U.S.A. [20]	NA	NA	NA	NA	NA	NA	NA	NA	NA	NA	NA	NA	NA	NA	NA	NA	NA	NA	NA	NA	NA	NA	NA	NA
Chua 2003 Singapore [46]	1	1	1	1	1	1	1	1	0	1	0	0	1	0	0	1	0	0	0	0	0	0	11	50
Wang 2008 Taiwan [50]	NA	NA	NA	NA	NA	NA	NA	NA	NA	NA	NA	NA	NA	NA	NA	NA	NA	NA	NA	NA	NA	NA	NA	NA
Mirkov 2009 New Zealand [51]	1	1	1	1	1	1	1	1	0	1	1	1	1	1	1	1	1	1	1	1	1	0	20	90.91
Chemello 2012 Spain [29,34]	1	1	1	1	1	1	1	1	1	1	1	1	1	1	1	1	1	1	1	1	1	1	22	100
Chen 2013 Singapore [52]	1	1	1	1	1	1	1	1	0	1	1	0	1	1	1	1	1	1	1	1	0	0	18	81.82
George 2017 India [44]	1	1	1	1	1	1	1	1	0	1	1	0	1	0	1	1	1	1	0	1	0	0	16	72.73
Lumbantobing 2017 Indonesia [48]	1	1	1	1	1	1	1	1	0	1	1	0	1	1	1	1	1	1	0	1	0	0	17	77.27
Alshamrani 2018 Saudi Arabia [47]	1	1	1	1	1	1	1	1	0	1	1	1	1	1	1	1	1	1	1	1	1	1	21	95.45
Manley 2020 U.S.A. [53]	1	1	1	1	1	1	1	1	1	1	1	1	1	1	1	1	1	1	1	1	1	1	22	100
Daifi 2021 U.S.A [54]	1	1	1	1	1	1	1	1	1	1	1	1	1	1	1	1	1		1	1	1	1	22	100
Peri 2022 Indonesia [45]	1	1	1	1	1	1	1	1	0	1	1	0	1	1	1	1	1	1	0	0	0	0	16	72.73
